# Targeting the TGFβ pathway in uterine carcinosarcoma

**DOI:** 10.15698/cst2020.11.234

**Published:** 2020-08-25

**Authors:** Shailendra Kumar Dhar Dwivedi, Geeta Rao, Anindya Dey, Megan Buechel, Yushan Zhang, Min Zhang, Da Yang, Priyabrata Mukherjee, Resham Bhattacharya

**Affiliations:** 1 Department of Obstetrics and Gynecology, University of Oklahoma Health Sciences Center, Oklahoma City, Oklahoma, USA.; 2 Department of Pathology, University of Oklahoma Health Sciences Center, Oklahoma City, Oklahoma, USA.; 3 Center for Pharmacogenetics, Department of Pharmaceutical Sciences, University of Pittsburgh, Pittsburgh, PA 15261, USA.; 4 Peggy and Charles Stephenson Cancer Center, University of Oklahoma Health Sciences Center, Oklahoma City, Oklahoma, USA.

**Keywords:** Uterine carcinosarcoma, Galunisertib, TGFβ1

## Abstract

Uterine carcinosarcoma (UCS) is a relatively infrequent, but extremely aggressive endometrial malignancy. Although surgery and chemotherapy have improved outcomes, overall survival (OS) remains dismal due to the lack of targeted therapy and biphasic (epithelial and mesenchymal) nature that renders the tumor aggressive and difficult to manage. Here we report a role of transforming growth factor-β (TGFβ) in maintaining epithelial to mesenchymal transition (EMT) phenotype and aggressiveness in UCS. Using a 3D-culture system, we evaluated the efficacy of the transforming growth factor-β receptor-I (TGFβR1) kinase inhibitor Galunisertib (GLT), alone and in combination with standard chemotherapeutic drugs used for the management of UCS. We demonstrate that GLT by inhibiting canonical and non-canonical signaling emanating from transforming growth factor-β1 (TGFβ1) reduces cellular viability, invasion, clonal growth and differentiation. Interestingly, GLT sensitizes UCS cells to chemotherapy both *in vitro* and in *in vivo* preclinical tumor model. Hence, targeting TGFβ signaling, in combination with standard chemotherapy, may be exploited as an important strategy to manage the clinically challenging UCS.

## INTRODUCTION

Uterine carcinosarcoma (UCS) is a highly aggressive tumor that constitutes 3-4% of uterine cancers [1, 2]. However, mortality from UCS is disproportionately higher (16.4%), among uterine malignancies [1, 3]. At diagnosis, UCS has a high rate of extrauterine metastasis and despite progresses noted with chemotherapy more than 70% of optimally resected stage III-IV patients recur within three years. Unfortunately, 80% of patients with advanced/recurrent disease succumb within two years [4–6]. UCS is characterized by biphasic tumors composed of epithelial and mesenchymal elements and demonstrated to be of monoclonal origin [2, 7–10], hence epithelial to mesenchymal transition (EMT) is considered a critical cellular process responsible for poor prognosis and therapy resistance [11, 12].

In non-malignant epithelial tissues, TGFβ1 plays an essential role in maintaining homeostasis by its ability to inhibit cell cycle progression and by promoting apoptosis [13–24]. The role of TGFβ1 in tumorigenesis is complex and its tumor promoting functions are closely linked to the initiation of an EMT program [25]. Aberrant TGFβ1 signaling in UCS [26] and other carcinomas of the breast and pancreas endow tumor cells with a selective advantage of enhanced motility and resistance to chemotherapeutics with an expansion of cancer-initiating stem-like cells [27–29]. Previously, we showed that the signaling proteins involved in the TGFβ pathway are expressed and functional in UCS [26]. Hence, there is an emergent need to address elevated TGFβ1 signaling using new therapeutic strategies in these malignancies. Here, we evaluated the impact of TGFβ1 signaling in a 3-dimensional (3D) Matrigel™ based culture system. The Matrigel™ based 3D culture system supports anchorage-independent growth and provides an acellular scaffold composed of collagen and other extracellular matrix (ECM) components, which, in part, recapitulate the tumor microenvironment [30].

Our results show that TGFβ1 induced both canonical and non-canonical signaling pathways in UCS that were associated with enhanced clonal growth, invasion and EMT. Treatment with the TGFβR1 inhibitor, Galunisertib (GLT) significantly inhibited all these phenotypes. Moreover, significant benefit from combining GLT with standard therapy, carboplatin and paclitaxel was demonstrated in a pre-clinical xenograft model of UCS. Therefore, combining a TGFβ inhibitor with standard chemotherapy may provide a promising therapeutic approach for the clinically challenging UCS.

## RESULTS

### Role of TGFβ1 in mediating an aggressive UCS phenotype

**Figure 1:**
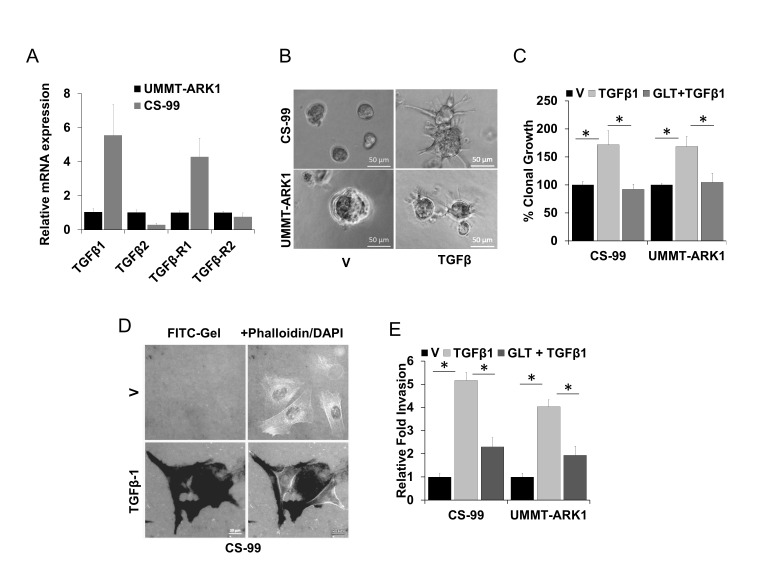
FIGURE 1: Role of TGFβ1 in mediating an aggressive UCS phenotype. **(A)** Relative mRNA expression of TGFβ1, TGFβ2, TGFβR1, and TGFβR2 in UMMT-ARK1 and CS-99 cells. Data are mean ± SE. **(B)** 1x10^4^ UMMT-ARK1 and CS-99 cells were grown in 3D culture, once the colonies were visible they were treated as indicated for 48 h and imaged. Representative images are shown. **(C)** 1x10^4^ UMMT-ARK1 and CS-99 cells were plated in Matrigel, treated with either control vehicle (V), TGFβ1 (5 ng/ml), or GLT (5 µM) + TGFβ1. Colonies were imaged and quantitated using Optronix Gelcount colony counter, % clonal growth compared to vehicle treated control are shown. **(D)** Representative images of CS-99 cells plated on Oregon Green 488 Gelatin–coated coverslips and treated with 5 ng/ml TGFβ1 for 18 h. The cells were fixed and the cellular F-Actin was stained with Alexa Fluor 555 Phalloidin and mounted in VECTASHIELD mounting medium containing DAPI, the dark areas in the left panels represent FITC-gelatin degradation. **(E)** Relative fold invasion derived from evaluation of <100 cells as shown in (D). Error bars represent standard deviation. *, P<0.05 was considered significant.

Based on our previous report on the significance of TGFβ signaling in UCS [26] and other studies demonstrating amplification of the TGFβ locus in UCS, [31] we evaluated the mRNA expression of TGFβ1, TGFβ2, TGFβR1 and TGFβR2 using reverse transcription quantitative real time polymerase chain reaction (RT-qPCR). mRNA for TGFβ1, TGFβ2, TGFβR1 and TGFβR2 could be detected in both the CS-99 and UMMT-ARK1 cell lines (**Fig. 1A**). To mimic in part the *in vivo* environment, CS-99 and UMMT-ARK1 were cultured in reduced growth factor Matrigels with or without TGFβ1 at 5 ng/ml and clonal growth, morphology and ECM degradation were determined. Following spheroid growth in the 3D-Matrigel, treatment with TGFβ1 for 48 h led to morphologically distinct cellular protrusions, characteristic for invasive mesenchymal cells (**Fig. 1B**). In clonal growth assays, TGFβ1 significantly stimulated growth that was inhibited by GLT (**Fig. 1C**). To determine the invasive potential, the FITC-gelatin degradation assay was performed. TGFβ1 induced robust ECM degradation in both cell lines that was mostly inhibited by treatment with GLT (**Fig. 1D, E**). These results demonstrate that the component of TGFβ signaling are expressed in UCS cell lines, TGFβ induces a significant increase in clonal and migratory potential and these TGFβ1 mediated phenotypes can be inhibited by the TGFβR1 kinase inhibitor GLT and support that such an approach may be useful to reduce aggressive properties of UCS.

### GLT inhibits canonical and non-canonical TGFβ1 signaling and increases cisplatin sensitivity

**Figure 2:**
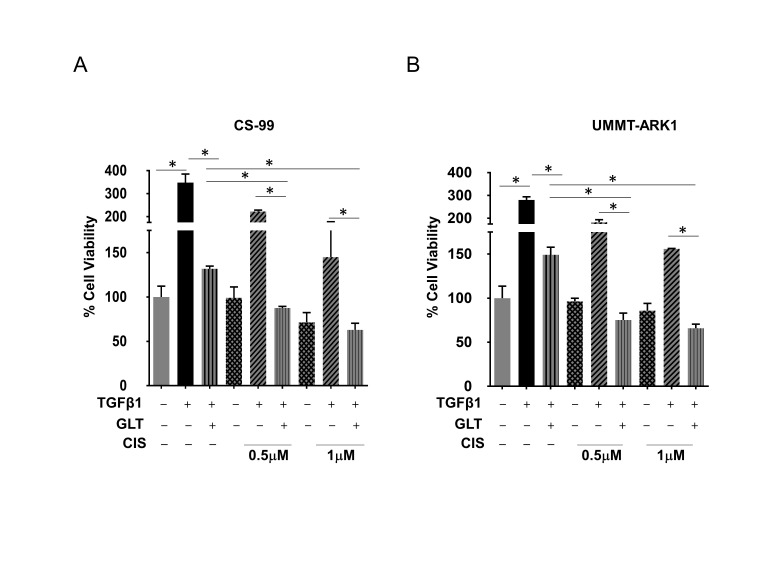
FIGURE 2: TGFβ1 in UCS cell proliferation and cisplatin sensitivity. **(A)** CS-99 and **(B)** UMMT-ARK1 cells were grown in Matrigel and treated as represented, post 72h treatment cell viability was evaluated using CellTiter-Glo® Luminescent Cell Viability Assay (Promega) and % cell viability compared to Vehicle (V) treated control is plotted. Data are mean ± SD of three independent experiments performed in triplicate. *, *P* < 0.05.

Since GLT inhibited TGFβ1 induced aggressive phenotypes, we next determined effects on cellular viability and signaling upon combination with cisplatin, a platinum drug used for the treatment of UCS patients. While GLT inhibited TGFβ1 induced cellular viability by ~62% and ~46% (compared to TGFβ1 treatment) in CS-99 and UMMT-ARK1 cells, respectively, combination of GLT with cisplatin and TGFβ1 treatment demonstrated a significant and dose-dependent decrease in cellular viability (**Fig. 2A, B**). Compared to the treatment with CIS+TGFβ1, GLT pretreatment reduced the viability by 60% compared to 0.5 µM CIS + TGFβ1 treatment and by 56% compared to 1 µM CIS + TGFβ1 treated CS-99 cells, and ~59% and ~58% decrease in cellular viability was observed in UMMT-ARK1 cells, respectively (**Fig. 2A, B**).

**Figure 3:**
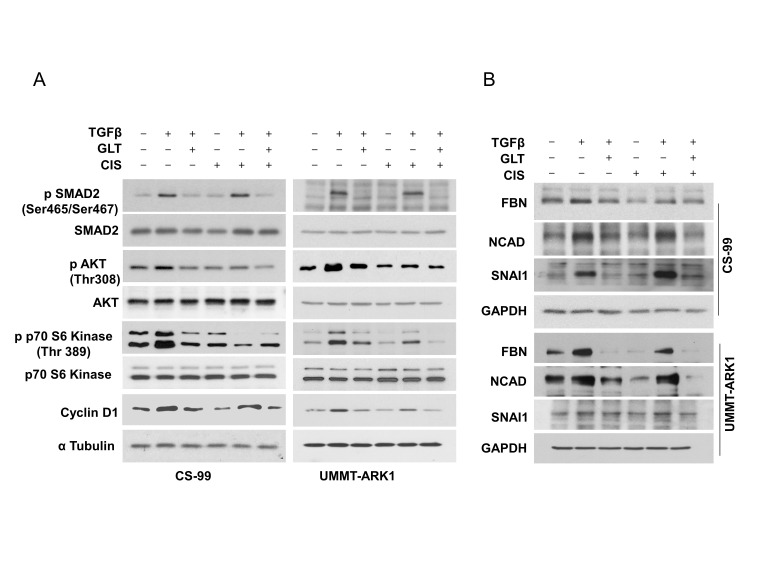
FIGURE 3: GLT inhibits canonical and non-canonical TGFβ signaling. CS-99 and UMMT-ARK1 cells were grown on a thin layer of Matrigel and treated with TGFβ1, GLT and Cisplatin (CIS, 2.5 µM) as represented, post 24 h of treatment, cell lysate was immunoblotted for **(A)** canonical and non-canonical markers of TGFβ1 signaling proteins along with Cyclin D1 as a marker for cell cycle progression and Tubulin as the loading control. **(B)** Effect of TGFβ1 and GLT + TGFβ1 treatment on the EMT markers (FBN – Fibronectin, NCAD - N-cadherin) were evaluated using immunoblotting, GAPDH was used as the loading control.

To determine the impact on associated signaling pathways we performed immunoblotting. Consistently, SMAD2 was activated by TGFβ1 and inhibited by GLT or upon combination with cisplatin (**Fig. 3A**). Similarly, AKT and p70S6 kinase were activated by TGFβ1 and inhibited upon combination with cisplatin and GLT (**Fig. 3A**). Together these results indicate that both canonical and non-canonical signaling is activated by TGFβ1 in these cell lines. Consequently, treatment with TGFβ1 increased Cyclin D1 levels, a proliferative marker that was inhibited by GLT or upon the combination with cisplatin (**Fig. 3A**). Additionally, treatment with TGFβ1 augmented expression of the mesenchymal markers Fibronectin (FBN), Snail Family Transcriptional Repressor 1 (SNAI1) and N-Cadherin (NCAD) that were inhibited by GLT or upon combination with cisplatin (**Fig. 3B**). Together these results corroborate that a decreased mesenchymal phenotype upon GLT treatment may be responsible for the increased sensitivity to cisplatin.

### GLT sensitizes tumor cells to chemotherapy in a mouse model

**Figure 4:**
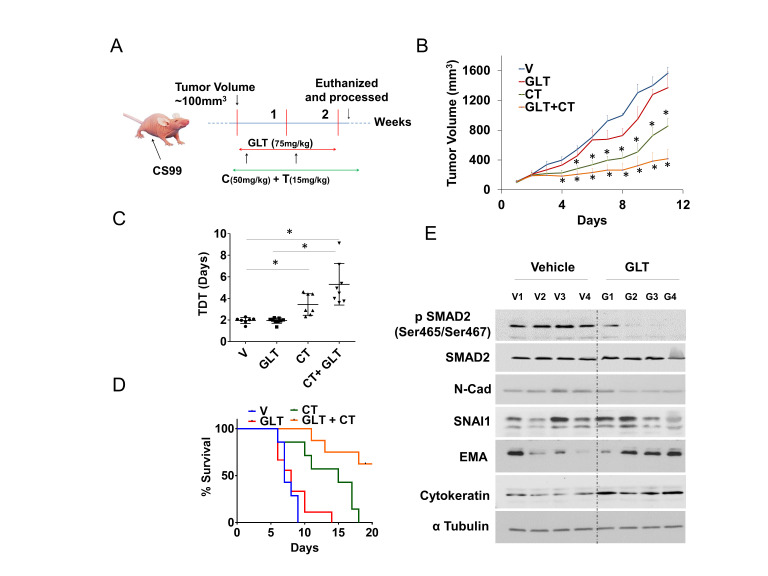
FIGURE 4: GLT sensitizes tumor cells to chemotherapy in a mouse model. **(A)** Female athymic nude mice were subcutaneously injected with CS-99 cells, once the tumor reached to ~100 mm^3^, tumor bearing mice were randomized into four groups (n = 7-10) and treated with either control vehicle (V), GLT, carboplatin & paclitaxel (CT), or GLT + CT. Treatment continued for two cycles (14 days), and mice were followed for survival. **(B)** Relative tumor volume normalized with V (vehicle) group. **(C)** Tumor doubling time (TDT) was calculated for each treatment groups according to Mehrara and colleagues [35]. Data are mean ± SD and *, P < 0.05 when comparing between indicated groups by one-way ANOVA **(D)** Percent survival was calculated by Kaplan–Meier method and P values determined by log-rank test based on the number of days the animals survived before the euthanization as per IACUC limits (humane endpoint). **(E)** Four tumor samples from vehicle treated control and GLT treated group were analyzed for the expression of TGFβ1 signaling and EMT markers using immunoblotting, alpha tubulin was used as loading control. Values are mean ±SD. *P<0.05.

A mouse xenograft model was developed using CS-99 cells [32] and was used to evaluate the efficacy of GLT *in vivo*. When tumor volume reached ~100 mm^3^, mice were randomized into four groups of 7-10 mice/group (**Fig. 4A**). The first group received vehicle only. The second group received 75 mg/kg GLT t twice daily by oral gavage for two weeks [33, 34]. The third group received intraperitoneal injections of 50 mg/kg carboplatin and 15 mg/kg paclitaxel (CT treatment), weekly for two weeks [32]. The fourth group received GLT along with carboplatin and paclitaxel at respective doses for two weeks. When the tumor burden reached humane endpoints (1500mm^3^) the animals were euthanized. Compared to the control vehicle group, a significant reduction in the tumor volume was observed with CT treatment (~45% reduction in tumor volume compared to vehicle treatment at endpoint). Combining GLT with CT further decreased UCS tumor volume (~73% reduction in tumor volume compared to vehicle treatment at endpoint; **Fig. 4B**). Tumor doubling time (TDT) was used to compare tumor growth rates over the entire experimental period [35]. Compared to the vehicle (TDT= ~1.9 days) or CT (TDT= ~3.4 days) treated group, TDT was significantly delayed in the CT + GLT treated group (~5.3 days; **Fig. 4C**) while there was no difference in the TDT of the group treated with GLT alone compared to the vehicle treated group. The median survival of the tumor bearing mice were similar in the vehicle and GLT treated groups, seven and eight days, respectively. In the CT treated group median survival was 16 days whereas, more than 50% of the mice survived beyond 20 days in the GLT+CT treated group (**Fig. 4D**). The decrease in phospho-SMAD2 accompanied with decreases in N-Cad, SNAI1 and increase in the EMA and the acidic/basic cytokeratin which are also known as types 1 and 2 keratin [36] suggest that the GLT treatment results in increased epithelial phenotype, thus making them more susceptible towards the chemotherapeutics. Hence, GLT treatment significantly inhibits TGFβ1 signaling, and along with CT increases survival and tumor doubling time in animals bearing UCS tumors. These results indicate that combining TGFβ1 inhibitors may sensitize UCS to the standard-of-care CT by inhibiting EMT and thus may be beneficial for patients.

## DISCUSSION

Lack of targeted drugs and aggressive nature of the tumor makes the management and therapy of UCS challenging. UCS was initially combined with other uterine sarcomas for treatment selection and clinical trial eligibility, however, recent studies have established that UCSs are not sarcomas [7, 8, 11]. Importantly, in UCS the acquired markers of EMT are upregulated and the TGFβ1 locus is amplified [31]. By investigating signaling in a 3D-culture system that in part recapitulates cellular interactions and the tumor microenvironment we have shown that TGFβ1 activates both canonical (SMAD dependent) and non-canonical (SMAD independent) signaling in UCS and induces extensive mesenchymal and invasive phenotype. Furthermore, in UCS preclinical models GLT treatment inhibits the mesenchymal phenotype and increases drug sensitivity.

The functional outcome of the TGFβ1 response is cell context-dependent and, in advanced carcinoma, enhances cellular invasion, promotes dissemination to distant tissues, increases angiogenesis, and promotes immune evasion [37–39]. In various cancers, including in UCS, pancreatic cancer, breast cancer, and colon cancer TGFβ-mediated canonical and non-canonical signaling pathways play key roles in EMT and drug resistance [40–49]. Interestingly, the TGFβ1 pathway has been implicated in metastatic processes and dramatically impacts the ability of tumor cells to spread throughout the body [39, 50–53]. However, to fully assess the consequence of TGFβ1 signaling in any malignancy it is important to appreciate the non-canonical pathways altered. For example, in NMuMG breast cancer cells TGFβ stimulated AKT and activated downstream effectors mTOR, P70S6K, and 4E-BP1, leading to increased cell size and proliferation that were associated with EMT [54]. Of the several non-canonical pathways that TGFβ potentiates [55, 56] in UCS, AKT and p70S6K were activated and subsequently inhibited by GLT. In an animal model, GLT treatment enhanced sensitivity to CT and prolonged survival. Therefore, in UCS the collaboration between the canonical and non-canonical pathways may be responsible for the aggressive phenotype.

The various candidate therapeutics that are currently being tested in clinical trials of UCS include Pazopanib, a multi-targeted tyrosine kinase inhibitor of the Vascular Endothelial Growth Factor Receptor (VEGFR), Platelet-derived Growth Factor Receptor and KIT Proto-Oncogene, Receptor Tyrosine Kinase combined with Gemcitabine (NCT02203760); AZD1775, a WEE1 G2 checkpoint kinase inhibitor (NCT03668340); a combination of Cabozantinib, an inhibitor of MET proto-oncogene, receptor tyrosine kinase (C-Met) and VEGFR2 and Nivolumab, a Programmed Cell Death 1 (PD-1) inhibitor with Ipilimumab, a monoclonal antibody targeting Cytotoxic T-Lymphocyte Associated Protein 4 (NCT04149275); a combination of Rucaparib, a PARP inhibitor, with Bevacizumab, a VEGF inhibitor and Atezolizumab, an anti-Programmed Cell Death 1 monoclonal antibody (NCT03694262). Also, the combination of Paclitaxel and Carboplatin is being tested in combination with Ifosfamide, an alkylating agent in recurrent UCS patients (NCT00954174). These therapeutic approaches impinge on pathways that are either anti-angiogenic, cytotoxic or target the immune system. Our present and prior results indicate that in UCS, TGFβ and EMT play a prominent role in disease progression and therefore targeting this pathway may be beneficial [26].

Several clinical inhibitors targeting the TGFβ1 pathway in cancer have been developed that are either ligand traps or block ligand-receptor interaction or inhibit the kinase activity of the TGFβ receptors [57]. Among these GLT, a small molecule selective inhibitor of the TGFβR1 kinase activity [58–60], is one of the more advanced drugs under clinical development. As reviewed in Neuzillet *et al.*, GLT is in Phase II clinical trials for pancreatic ductal adenocarcinoma, hepatocellular carcinoma, glioma and glioblastoma [57]. In conclusion, our results clearly suggest that targeting TGFβ1 using GLT may be exploited as an important therapeutic approach to reduce tumor growth, EMT and to overcome therapy resistance in UCS.

## MATERIALS AND METHODS

### Cell culture

The human UCS cell line UMMT-ARK1 was derived from uterine carcinosarcomas and was a kind gift from Dr. Alessandro Santin [61] (Department of Obstetrics, Gynecology & Reproductive Sciences, Yale University School of Medicine, New Haven, CT). UMMT-ARK1 cells have been established from the uterine carcinosarcoma specimens and characterized by the presence of vimentin and cytokeratin AE1/AE3. CS-99, developed from uterine carcinosarcoma was a kind gift from Dr. Jason Somarelli; Duke University Medical Center [26, 62]. The mesenchymal phenotype was evidenced immunohistochemically by strong vimentin and moderate SM-actin, which was similar to the sarcomatous component of the primary tumor. Epithelial membrane antigen (EMA) was moderately expressed whereas other markers including pan cytokeration (CK), CK 5/6, CK 34, epidermal growth factor receptor were also expressed in CS-99. UMMT-ARK1 and CS-99 cells were maintained in RPMI 1640 and DMEM respectively, supplemented with 10% heat inactivated FBS (Fisher Scientific), 100 unit penicillin and 100 μg streptomycin/ml (Invitrogen). For 3D culture, the cells were grown on growth factor reduced Matrigel (Corning).

### RNA isolation, reverse transcription and qPCR

To evaluate the mRNA expression of TGFβ1, TGFβ2, TGFβR1, and TGFβR2 in UMMT-ARK1 and CS-99 cells, total RNA was isolated using Quick-RNA™ Miniprep Kit (Zymo Research) quantified, 1 μg RNA was reverse transcribed (iScript cDNA Synthesis Kit, BioRad) and quantitative polymerase chain reaction was performed using iTaq Universal SYBR Green (BioRad). Relative mRNA expression levels were calculated using the comparative ΔΔCt method with B2M as the normalizer.

### Cell viability assay

The viability of UCS cells was determined using the CellTiter-Glo® Luminescent Cell Viability Assay (Promega). For proliferation assay, 3x10^3^ cells were mixed in growth medium containing 1 mg/ml of Matrigel on top of 1mg/ml Matrigel base layer. Cells were treated as required, in case of GLT + TGFβ1 treatment cells were pre-treated with GLT for one hour and then treated with TGFβ1. Post 72h of treatment viability was measured using luminescence based CellTiter-Glo® which measures the ATP content of the cells.

### Clonal growth

1×10^3^ UMMT-ARK1 or CS-99 cells were mixed in 1 mg/ml Matrigel and were seeded on top of 3mg/ml Matrigel in each well of the 12-well plate. Cells were treated either with vehicle (CTL), TGFβ1 (5 ng/ml) or GLT (5 µM) + TGFβ1. In GLT + TGFβ1 group cells were pretreated with GLT (1 h before TGFβ1 treatment). Colonies were visible in around 5 in CS-99 and in 7 days in UMMT-ARK1. Colonies were imaged and counted using an Optronix GelCount colony counter (Abingdon OX14 4SA, United Kingdom).

### Gelatin degradation assay

Oregon Green^®^ 488 fluorophore-conjugated gelatin coated coverslips were prepared as described previously [26]. For degradation assay, 2x10^4^ cells were seeded in each well of a 24-well plate containing Oregon Green^®^ 488 fluorophore-conjugated gelatin coated coverslips. Cells were treated after 8 hours and 18 h post-treatment cells were fixed in 4% Paraformaldehyde (PFA) and stained with Alexa Fluor^®^ 555 Phalloidin (Life Technologies, Rockford, IL, USA) following manufacturer's protocol and mounted with DAPI containing VECTASHIELD^®^ mounting medium (Vector Laboratories) [63]. Relative migratory potential of the cells were analyzed using the Zeiss Axio-Observer Z1 microscope (Göttingen, Germany). Cells that degraded the ECM at focal adhesions (FA) sites were scored as positive, and more than 100 random cells were quantified and statistical significance was analyzed by two-tailed *t* test.

### Western blotting

For immunoblotting cells were grown on Matrigel and treated as represented in the image. In the case of GLT combination treatments, cells were pretreated with GLT and then with TGFβ1. Post 24 h of treatment, cells were trypsinized and washed with cold PBS to dissolve Matrigel. The cells were lysed in protease and phosphatase inhibitor (Thermo-Fisher) containing RIPA (Boston Bioproducts) buffer. The protein content of the lysate were quantified using BCA assay (Thermo Fisher Scientific) and western blot was performed with equal protein. Membranes were blocked for 1 h at room temperature (5% nonfat milk) followed by overnight incubation with primary antibody. Following primary antibodies were used – p Smad-2 (#18338), Smad-2 (#5339), SNAI1 (#3879), Cyclin D1 (#55506), (Cell signaling Technology), Fibronectin (#610077), N-Cad (#610921)(BD Biosciences), GAPDH (#G9295), and α tubulin (#T5201) (Sigma), Acidic and basic Cytokeratin AE1/AE3 (#MAB3412) (Millipore) and EMA (#GA62961) (Dako). Secondary antibodies (from sigma) were used at a concentration of 1:10,000. Equal loading was verified by immunoblotting with GAPDH or αTubulin.

### Mouse xenograft model

The animal studies were approved by the University of Oklahoma Animal Facility under the guidance of IACUC and were performed as described previously [32]. Briefly, female athymic nude mice (NCr-nu; 6–8 weeks old, ENVIGO Laboratories) were subcutaneously injected with CS-99 cells (1×10^6^/100 μL in Opti-MEM). Once the tumor volume reached approximately 100 mm^3^, mice were randomized to four different groups receiving vehicle, GLT (Eli-Lilly and company) or standard of care drugs carboplatin (Hospira, Inc.) and paclitaxel (Actavis Pharma, Inc.; CT) or GLT+ carboplatin and paclitaxel. GLT was administered orally at 75 mg/kg body weight twice daily for 14 days. Carboplatin and paclitaxel were given once weekly by intraperitoneal injection at 50 and 15 mg/kg body weight, respectively [32]. After two cycles of treatment, mice were followed for tumor growth and euthanized according to IACUC limits (~1500 mm^3^). Tumor doubling time (DT) was calculated according to Mehrara and colleagues using the equation DT = LN (2)/SGR, and SGR (specific growth rate) = ln(*V*_2_/*V*_1_)/(*t*_2_−*t*_1_) [35]. In this experiment, we have utilized 7 mice in the vehicle-treated group, 9 mice in GLT group, 7 mice in C + T group and 9 mice in GLT+CT group, respectively.

### Data analysis and statistics

Quantification and statistics are detailed in the Figure legends. Data are expressed as mean ± SD unless otherwise noted. One-way ANOVA was performed to compare the mean among three or more groups, and the Student *t*-test was performed for comparison between two groups. Survival analysis was performed using Kaplan–Meier method and log-rank analysis by the GraphPad Prism 6 software.

## Abbreviations:

CT: – carboplatin/paclitaxel treatment;
ECM: – extracellular matrix;
EMT: – epithelial to mesenchymal transition;
GLT: – Galunisertib;
TGF: – transforming growth factor;
TDT: – tumor doubling time;
UCS: – uterine carcinosarcoma.
